# Insights Into Associations Between Oral and General Health Outcomes of Nursing Home Residents Based on InterRAI Data: Cross-Sectional Study

**DOI:** 10.2196/72308

**Published:** 2025-10-17

**Authors:** Emilie Schoebrechts, Johanna de Almeida Mello, Patricia Ann Ivonne Vandenbulcke, Hein Petrus Johannes van Hout, Jan De Lepeleire, Anja Declercq, Dominique Declerck, Joke Duyck

**Affiliations:** 1 Population Studies in Oral Health, Department of Oral Health Sciences, KU Leuven, Kapucijnenvoer 7, Leuven, 3000, Belgium; 2 LUCAS, Center for Care Research and Consultancy, KU Leuven, Leuven, Belgium; 3 Departments of General Practice and Medicine for Older People, Amsterdam UMC, Vrije Universiteit Amsterdam, Amsterdam, The Netherlands; 4 Amsterdam Public Health Research Institute, Aging & Later Life Program, Amsterdam UMC, Amsterdam, The Netherlands; 5 Department of Public Health and Primary Care, Academic Center for General Practice, KU Leuven, Leuven, Belgium; 6 CeSO, Center for Sociological Research, KU Leuven, Leuven, Belgium

**Keywords:** advance care planning, long-term care, geriatric conditions, oral health, oral care, geriatric assessment

## Abstract

**Background:**

Oral health of nursing home residents is generally poor, largely due to age-related conditions that increase their vulnerability and make them more dependent on others for oral care. Poor oral health can significantly impact general health and well-being, highlighting the important role of caregivers in preventing, detecting, and addressing residents’ oral health problems in time. The interRAI Suite of instruments, widely used for comprehensive health assessments, offers the opportunity for nondental caregivers to assess oral health as part of general health and well-being and facilitates the integration of oral health in general care planning.

**Objective:**

Based on interRAI data, this study explored the associations between oral health and general health outcomes of nursing home residents in Flanders (Belgium) and the Netherlands.

**Methods:**

This cross-sectional study included baseline interRAI assessments of 2362 Flemish and Dutch residents aged 65 years and older, collected by caregivers (eg, nurses, nurse aids) between October 2020 and February 2024. Validated outcome scales (eg, Activities of Daily Living Hierarchy Scale, Cognitive Performance Scale, and Depression Rating Scale), health conditions such as diabetes and low weight (BMI ≤19), and family support and participation in social activities, included in the interRAI instrument, provided information on residents’ general health and well-being. Oral health was defined according to the 9 items of the optimized Oral Health Screener (OHS) for use in the interRAI instruments. Adjusted logistic regression models were used to explore the associations between oral and general health outcomes.

**Results:**

Oral health problems were interconnected, with conditions in one oral structure impacting the health of others. When controlling for confounding variables, dependency on others for personal care and hygiene was significantly associated with poor oral hygiene (odds ratio [OR] 1.7, 95% CI 1.1‐2.4), chewing difficulties (OR 2.2, 95% CI 1.3‐3.5), compromised teeth (OR 1.7, 95% CI 1.1‐2.5), and the need for dental referral (OR 1.8, 95% CI 1.4‐2.3). Natural dentition and gender had a significant impact on oral health status. Low weight was associated with poor chewing function (OR 2.0, 95% CI 1.1‐3.6) and dry mouth (OR 1.8, 95% CI 1.0‐3.2), both associated with oral discomfort or pain (OR 6.8, 95% CI 3.3‐14.0 and OR 3.5, 95% CI 1.8‐6.9, respectively). Family support was identified as a facilitator in mitigating oral health problems.

**Conclusions:**

The interRAI instrument, including the OHS, is a valuable tool for monitoring residents’ health and identifying areas requiring additional support. The results demonstrated the importance of prioritizing oral health as an integral component of comprehensive care. Recognizing the interconnection between oral health and other health conditions, including indicators of well-being, can guide caregivers and other health care professionals in incorporating preventive and supportive oral care practices into daily care routines, contributing to improved resident health and well-being outcomes.

## Introduction

Oral diseases are among the most widespread noncommunicable diseases, affecting nearly half of the population worldwide, with older adults among the most vulnerable persons [1]. Common oral diseases and conditions in older persons include dental caries, periodontitis, tooth loss, and dry mouth [2]. Although most oral diseases are largely preventable with adequate daily care and regular dental checkups, older adults face several challenges, making it difficult to maintain good oral health. These challenges include a reduced ability to self-care due to compromised general health, polypharmacy, difficult accessibility, and inadequate availability of dental care [3,4].

Suboptimal oral health can have major consequences for general health and quality of life and vice versa [5]. Research has shown a bidirectional relationship between periodontitis and systemic diseases such as diabetes and cardiovascular disease [6]. Individuals with cognitive impairment may have difficulty understanding the importance of oral health and maintaining consistent hygiene routines [3,7]. Conversely, tooth loss has been associated with cognitive decline, due to reduced chewing ability [8]. Wearing dentures at night can increase the risk of pneumonia due to accumulated plaque levels [3]. In addition, certain types of medications can reduce saliva production, increasing the risk of oral health problems [9]. Poor oral health can also lower a person’s self-esteem, leading to social isolation and depressive symptoms [3,10]. Conversely, depression and other mental health conditions often result in neglected oral hygiene due to a lack of motivation or energy, which worsens oral health [3,10]. Given the complex interactions between oral and general health, it is important to address oral health problems in time to prevent further health deterioration.

Through regular oral health assessments, caregivers can help improve oral health of older adults by enabling prevention, early detection, and timely treatment of oral health problems [11,12]. In this regard, the interRAI Suite of instruments is useful due to its holistic approach and wide range of applications [13].

The interRAI Suite consists of comprehensive web-based assessment instruments for caregivers to collect information in a standardized way from care-dependent persons on key factors including physical and cognitive function, psychosocial factors, clinical situation, social support, and so on. These instruments have been introduced in more than 35 countries and are compatible across different care settings. The interRAI Suite provides reliable and high-quality data to improve continuity of care, promote a person-centered approach, and enhance the ability to assess clinical outcomes. The integration of collaborative action points (CAPs), which are trigger algorithms to identify care needs and the risk of adverse outcomes, facilitates collaborative decision-making and helps prevent health deterioration and improve quality of life. Since the interRAI instrument for use in long-term care facilities (LTCFs) (eg, nursing homes) includes oral health as an integral part of general health and well-being, it has the potential to integrate oral care into general care planning [13,14].

However, research has shown that the oral health section within version 9 of the interRAI instrument is incomplete and has limited validity [15]. Replacing the oral health section in the interRAI LTCF with an optimized oral health section may allow caregivers to more effectively identify oral health problems, while also considering the other non–oral health conditions indicated in the assessments that may serve as risk factors [16].

Therefore, an optimized oral health section, the ohr-interRAI, was developed and recently identified in a systematic review as the most appropriate oral health instrument for nondental caregivers to evaluate older adults’ oral health [12,15,17]. Based on a Delphi study with 53 oral health experts from 34 countries, further improvements were made, resulting in the Oral Health Screener (OHS) for use in the interRAI instruments, which takes into account national and cultural differences that may exist regarding oral health, oral care, and oral behavior [18].

The OHS consists of 9 items: chewing function, discomfort or pain in the mouth, dry mouth, hygiene of removable dentures, oral hygiene, teeth, gums, tongue, and palate and inner surface of cheeks and lips. Based on the assessment of these items, the CAP *oral hygiene* and the CAP *referral to a dentist* will be activated when help with daily oral hygiene and a referral to a dentist is needed, respectively. To help caregivers identify oral health problems, photographs, guidelines on the general assessment, items and CAPs, and instruction videos are available [17,18].

Although not yet officially included in the global interRAI Suite of instruments, the OHS is already in use in nursing homes in Flanders (Belgium) and in the Netherlands. Since the interRAI LTCF instrument collects data on oral health and general health, including well-being, from residents, it allows for assessing the associations between these outcomes. This study is the first to explore such associations using the OHS. Given the limited validity of the current oral health section and the widespread use of the interRAI instruments, this study aims to support the implementation of the OHS in the interRAI instruments and to strengthen the interRAI’s overarching goals. Understanding the links between oral health and other health problems can facilitate early intervention and comprehensive care planning, thereby improving residents’ quality of life. In addition, recognizing these associations contributes to prevention, supporting cost-effective health care, and guides the development of policies and preventive programs targeting oral and general health in older populations.

## Methods

### Study Design and Inclusion Criteria

This cross-sectional study included interRAI LTCF baseline data from persons aged 65 years and older living in nursing homes in Flanders (Belgium) and in the Netherlands. The data were collected by professional caregivers (nurses, physiotherapists, occupational therapists, etc) using the interRAI LTCF instrument, including the OHS, to assess residents’ oral and general health status. This study differs from a previous study by Schoebrechts et al [19], reporting the prevalence of oral health problems identified using the OHS, by including data from a larger number of residents and examining associations with other health and well-being indicators. All data were collected between October 2020 and February 2024.

### Ethical Considerations

This multicenter study was approved by the Belgian Privacy Commission and the Ethics Committee Research UZ/KU Leuven (B3222021000448). All participants or their family members gave informed consent for the use of their data for research purposes. To ensure privacy and confidentiality, all data were pseudonymized and data access was limited to the research team. No compensation was provided for participation.

### Outcomes

#### Oral Health

Using the OHS, the assessment of chewing function, discomfort or pain in the mouth, and dry mouth requires a conversation or observation during meals, while the assessment of *hygiene of removable dentures*, *oral hygiene*, *teeth*, *gums*, *tongue,* and *palate and inner surface of cheeks and lips* requires a visual inspection in the mouth. These items are assessed on a scale that distinguishes between acceptable and unacceptable conditions, with photographs available for the 6 items requiring a visual inspection. In addition, caregivers have the option to indicate whether the assessment is not possible (eg, if persons resist) or not applicable (eg, no dentures). The CAP *oral hygiene* and the CAP *referral to a dentist* are activated when help with daily oral hygiene and a referral to a dentist are needed, respectively.

#### General Health

General health, including well-being, outcomes in the interRAI instrument were selected based on previous research indicating their relevance to oral health. The interRAI instrument includes internationally validated outcome scales to evaluate a person’s clinical status, which are automatically calculated based on a number of individual items [20]. The following scales were included in the analyses. They were dichotomized using validated cutoff values, with higher scores indicating greater severity.

The Activities of Daily Living Hierarchy (ADLH) Scale, ranging from 0 to 6 (cutoff ≥3), refers to the degree of dependence on personal care, based on the items related to personal hygiene, toilet use, locomotion, and eating [21]. The Cognitive Performance Scale (CPS), ranging from 0 to 6 (cutoff ≥3), represents a person’s cognitive status, including cognitive skills in daily decision-making, short-term memory, procedural memory, ability to make self-understood, and eating self-performance [22]. The Depression Rating Scale (DRS), ranging from 0 to 14 (cutoff ≥3), represents the presence of depressive symptoms based on several items such as making negative statements, expressions of unrealistic fears, and repetitive health complaints or crying [23]. The Changes in Health, End-Stage Disease, Signs, and Symptoms (CHESS) Scale, ranging from 0 to 5 (cutoff ≥3), demonstrates a person’s health instability and is a strong predictor of mortality. The scale is based on several items such as changes in decision-making and activities of daily living status, dyspnea, dehydration, and weight loss [24]. The Communication Scale has a range from 0 to 8 (cutoff ≥4) and is calculated based on residents’ ability to make themselves understood and to understand others [25]. The Revised Index of Social Engagement, ranging from 0 to 6 (cutoff ≥3), is calculated from items describing an individual’s sense of initiative and social involvement in the nursing home. Unlike the other scales, a higher Revised Index of Social Engagement score indicates a positive outcome [26].

In addition, residents’ general health was evaluated based on the presence of the following health diseases recorded in the interRAI instrument: dementia, diabetes mellitus, congestive heart failure, chronic obstructive pulmonary disease (COPD), Parkinson disease, anxiety, bipolar disorder, schizophrenia, and pneumonia. Other health conditions included aspiration, reflux, low weight (BMI ≤19), and polypharmacy (≥10 medications). Information on residents’ mood or behavior status and psychosocial well-being was collected by including the interRAI items *resistance to care*, *reduced social interactions*, *strong and supportive relationships with family*, and the CAP *behavior*. This CAP is calculated based on residents’ wandering behavior, verbal and physical abusive attitude, socially inappropriate or disruptive behavior, and resistance to care. In addition, information in the interRAI instrument such as *extensive to total dependency for personal hygiene*, *modified mode of nutritional intake* (due to not swallowing all types of food), and *severe vision impairment in adequate light* was included in the analyses as well as the item on having a dental checkup in the last year and information on a physician visit in the last 14 days. All items were dichotomized.

#### Covariates

The following variables were considered covariates: age (divided into 3 groups: 65‐75, 76‐85, and 86+ years), sex (female and male), smoking (smoker and nonsmoker), presence of teeth (based on the *not applicable* response option of item *teeth*), and presence of dentures (based on the *not applicable* response option of item *denture hygiene*).

### Missingness Information

Analysis of missing values showed that they were random, which means that the probability of a missing value did not depend on the item itself or on certain characteristics of the assessors [19,27]. Possible causes for incomplete assessments with the interRAI instrument are discussed in detail by Vanneste et al [28].

### Data Analyses

Descriptive statistics were used to illustrate the characteristics (eg, demographic information and oral and general health status) of the residents. Categorical variables were summarized as absolute numbers and percentages, and continuous variables were presented as means and accompanying standard deviations.

For bivariate analyses, the oral health items, originally assessed using 3 or 4 response options, were dichotomized considering only the options when an assessment was possible (ie, acceptable or unacceptable condition). Unadjusted logistic regression models were performed to explore associations between the oral health–related and the selected general health–related variables. These analyses provided a preliminary understanding of the direct associations between the individual variables. Subsequently, multivariable logistic regression models were developed for each oral health item adjusted for oral and general health variables. Each model included a fixed set of explanatory variables (*ADLH/extensive to total dependency for personal hygiene*, *CPS*, *DRS*, *CHESS*, *Communication Scale*, *strong and supportive relationships with family,* and covariates), together with variables identified as significant in the bivariate analyses or supported by previous research. The variables considered as covariates were included in the regression models to control for potential confounding variables. *Denture hygiene*, *oral hygiene,* and *teeth* were excluded as explanatory variables in the models because these variables were assessed only in residents with dentures and natural teeth, respectively. Including these variables would have restricted the models to a limited subgroup of residents without dentures, without natural teeth, or without both. To address collinearity, variables measuring the same construct or part of another item (eg, *extensive to total dependency for personal hygiene* and *ADLH scale, resistance to care* and CAP *behavior*, and *presence of teeth* and *teeth*) were excluded from the models. *Extensive to total dependency for personal hygiene* variable was included instead of the ADLH Scale in the models for *denture hygiene*, *oral hygiene*, and CAP *oral hygiene*, as it may provide a better indication of these items’ status. The associations between the variables were expressed using odds ratios (ORs) and 95% CIs. All statistical analyses were performed using IBM SPSS Statistics (version 28.0.1.1; IBM) and SAS Enterprise Guide (version 8.1; SAS Institute). A *P* value of <.05 was considered statistically significant.

## Results

### Baseline Characteristics

The data consisted of 2362 baseline assessments (ie, first assessments of the residents using the interRAI instrument, including the OHS), 789 from 12 Flemish nursing homes and 1573 from 26 Dutch nursing homes. The mean age of the residents was 82.17 (SD 7.53) years and most residents were female (1567/2362, 66.3%). About 8.0% (180/2346) of the residents were daily smokers (Table 1).

**Table 1.** Baseline characteristics of the participating nursing home residents.

Characteristics	Value (N=2362)
Age (years), mean (SD)	82.17 (7.53)
Age range (years), n (%)^a^	
65‐75	633 (26.8)
76‐85	1121 (47.5)
86+	608 (25.7)
Sex, n (%)^a^	
Female	1567 (66.3)
Male	795 (33.7)
Daily smoker, n (%)^a^	180 (7.7)

a Specific outcome per item.

### Oral Health

Table 2 shows the distribution of the oral health conditions in the sample. Of the 3 self-reported items, chewing problems (290/2254, 12.9%) were the most reported condition by the nursing home residents. About 10.0% (226/2214) of the residents indicated having a dry mouth, and 4.0% (89/2219) reported discomfort or pain in the mouth. The condition of the teeth could be assessed in 49.8% (1147/2301) of the nursing home residents. This was found to be compromised in 25.6% (294/1147) of these residents. The second most commonly observed oral health problem was poor oral hygiene, identified in 20.6% (313/1519) of the residents with assessable oral hygiene (1519/2297, 66.1%). Hygiene of removable dentures could be assessed in 64.9% (1499/2310) of the participants with dentures and was found to be poor in 9.1% (136/1499). Gum problems were observed in 7.7% (155/2006) of the residents, problems with the tongue were observed in 4.0% (87/2057), and problems with the palate and inner surface of cheeks and lips were also observed in about 4.0% (76/1959) of the residents.

**Table 2.** The oral health status of the participating nursing home residents.

Oral health items		Residents, n/N^a^ (%)
Chewing function (n=2323)		
Assessed		2254/2323 (97.0)
Good/acceptable		1964/2254 (87.1)
Poor/unacceptable		290/2254 (12.9)
Not assessed		69/2323 (3.0)
Cannot be assessed^b^		42/69 (60.9)
Not applicable (eg, blended food due to swallowing difficulties and tube feeding)		27/69 (39.1)
Missing oral health data		39/2362 (1.6)
Discomfort or pain in the mouth (n=2346)		
Can be assessed		2219/2346 (94.6)
No		2130/2219 (96.0)
Yes		89/2219 (4.0)
Not assessed		127/2346 (5.4)
Cannot be assessed		127/127 (100)
Missing oral health data		16/2362 (0.7)
Dry mouth (n=2346)		
Can be assessed		2214/2346 (94.4)
No		1988/2214 (89.8)
Yes		226/2214 (10.2)
Not assessed		132/2346 (5.6)
Cannot be assessed		132/132 (100)
Missing oral health data		16/2362 (0.7)
Hygiene of removable dentures (n=2310)		
Can be assessed		1499/2310 (64.9)
Good /acceptable		1363/1499 (90.9)
Poor/unacceptable		136/1499 (9.1)
Not assessed		811/2310 (35.1)
Cannot be assessed		183/811 (22.6)
Not applicable (no dentures)		628/811 (77.4)
Missing oral health data		52/2362 (2.2)
Oral hygiene (n=2297)		
Can be assessed		1519/2297 (66.1)
Good/acceptable		1206/1519 (79.4)
Poor/unacceptable		313/1519 (20.6)
Not assessed		778/2297 (33.9)
Cannot be assessed		217/778 (27.9)
Not applicable (no teeth or denture retainers)		561/778 (72.1)
* *Missing oral health data		65/2362 (2.7)
Teeth (n=2301)		
Can be assessed		1147/2301 (49.8)
Good/acceptable		853/1147 (74.4)
Poor/unacceptable		294/1147 (25.6)
Not assessed		1154/2301 (50.2)
Cannot be assessed		193/1154 (16.7)
Not applicable (no teeth and no root remnants)		961/1154 (83.3)
Missing oral health data		61/2362 (2.6)
Gums (n=2295)		
Can be assessed		2006/2295 (87.4)
Good/acceptable		1851/2006 (92.3)
Poor/unacceptable		155/2006 (7.7)
Not assessed		289/2295 (12.6)
Cannot be assessed		289/289 (100)
Missing oral health data		67/2362 (2.8)
Tongue (n=2308)		
Can be assessed		2057/2308 (89.1)
Good/acceptable		1970/2057 (95.8)
Poor/unacceptable		87/2057 (4.2)
Not assessed		251/2308 (10.9)
Cannot be assessed		251/251 (100)
Missing oral health data		54/2362 (2.3)
Palate and inner surface of cheeks and lips (n=2299)		
Can be assessed		1959/2299 (85.2)
Good/acceptable		1883/1959 (96.1)
Poor/unacceptable		76/1959 (3.9)
Not assessed		340/2299 (14.8)
Cannot be assessed		340/340 (100)
Missing oral health data		63/2362 (2.7)

a Data included in the model/total available oral health data.

b “Cannot be assessed” when the item could not be assessed adequately, for example, due to resistant behavior or cognitive impairment.

Based on the results of the assessments with the OHS, the CAP *oral hygiene* was activated in 16.8% (384/2289), indicating a need for help with daily oral care, and the CAP *referral to a dentist* was triggered in 27.5% (611/2221), indicating a need for dental treatment. In addition, caregivers indicated a dental checkup in the last year in 56.5% (1319/2333) of the nursing home residents.

### General Health

Analyses of the outcome scales revealed that the majority of the residents were dependent on others for activities of daily living (1276/2351, 54.3%) and were cognitively impaired (1335/2341, 57.0%). Symptoms of depression were found in 36.7% (862/2346) and poor communication in 24.3% (5272/2351). Based on the CHESS Scale, health instability was present in 5.5% (126/2296) of the nursing home residents. Most residents (1902/2337, 81.4%) showed initiative and social involvement in the nursing home.

About 30.0% (706/2345) of the residents were experiencing dementia, 21.6% (506/2344) were experiencing diabetes, and 27.0% (634/2346) had behavior problems. The use of polypharmacy, indicated by taking 10 or more medications, was noted in 30.7% (721/2345). Strong and supportive relationships with family were reported by 88.2% (2070/2346) of the residents. More information on residents’ general health and well-being is shown in Table 3.

**Table 3.** The general health status of the participating nursing home residents.

	Residents (N=2362), n/N^a^ (%)
Scales	
Activities of Daily Living Hierarchy (0‐6) ≥3	1276/2351 (54.3)
Cognitive Performance Scale (0‐6) ≥3	1335/2341 (57.0)
Depression Rating Scale (0‐14) ≥3	862/2346 (36.7)
Changes in Health, End-Stage Disease and Symptoms and Signs (0‐5) ≥3	126/2296 (5.5)
Communication (0‐8) ≥4	5272/2351 (24.3)
Revised Index of Social Engagement (0‐6) ≥3	1902/2337 (81.4)
Diseases/disorders	
Anxiety	278/2350 (11.8)
Schizophrenia	28/2348 (1.2)
Bipolar	33/2349 (1.4)
Pneumonia	42/2348 (1.8)
Aspiration	187/2350 (8.0)
Reflux	74/2353 (3.1)
Dementia	706/2345 (30.1)
Diabetes mellitus	506/2344 (21.6)
Parkinson	157/2350 (6.7)
Chronic obstructive pulmonary disease	213/2344 (9.1)
Congestive heart failure	539/2342 (23.0)
Polypharmacy (≥10 medications)	721/2345 (30.7)
Low weight (BMI ≤19)	274/2257 (12.1)
Modified mode of nutritional intake	437/2356 (18.5)
Behavior problems	634/2346 (27.0)
Resistance to care	456/2349 (19.4)
Extensive to total dependency for personal hygiene	1139/2353 (48.4)
Severe vision impairment in adequate light	78/2348 (3.3)
Reduced social interactions	680/2347 (29.0)
Strong and supportive relationships with family	2070/2346 (88.2)
Physician visit in the last 14 days	901/2315 (38.9)
Dental checkup in last year	1319/2333 (56.5)

a Specific outcome per item/total available data per item.

#### Associations Between Oral Health and General Health Outcomes

Table S1 in Multimedia Appendix 1 and Tables 4 and 5 show the results of the bivariate analyses and the associated factors for each oral health item in the adjusted multivariable regression models, respectively.

**Table 4.** Factors associated with each oral health item (reference category: acceptable condition) in the adjusted logistic models.

		Chewing function (1564/2254)^a^		Discomfort or pain in the mouth (1609/2219)^a^		Dry mouth (1559/2214)^a^		Denture hygiene (1206/1499)^a^		Oral hygiene (1171/1519)^a^	
		OR^b^	95% CI	OR	95% CI	OR	95% CI	OR	95% CI	OR	95% CI
Age (years; 65-75: reference category)											
76‐85		1.93	1.15‐3.25^c^	0.88	0.42‐1.84	0.77	0.49‐1.21	1.11	0.63‐1.96	0.95	0.63‐1.45
>85		1.93	1.08‐3.44^c^	1.04	0.45‐2.39	1.14	0.69‐1.89	0.94	(0.48‐1.83	0.97	0.58‐1.62
Sex (male: reference category)											
Female		0.65	0.42‐1.02	0.73	(0.37‐1.44	0.68	0.45‐1.03	1.33	0.84‐2.11	1.57	1.08‐2.28^c^
Smoker		1.09	0.48‐2.47	0.45	0.11‐1.74	1.12	0.58‐2.17	1.53	0.73‐3.19	1.47	0.77‐2.81
ADLH^d^ Scale ≥3		2.16	1.34‐3.49^e^	0.75	0.40‐1.38	1.06	0.72‐1.56	—^f^	—	—	—
Extensive to total dependency for personal hygiene		—	—	—	—	—	—	1.39	0.87‐2.22	1.65	1.13‐2.41^c^
CPS^g^ ≥3		1.29	0.80‐2.07	0.91	0.46‐1.81	0.54	0.36‐0.82^e^	1.05	0.63‐1.75	0.98	[0.65‐1.47
DRS^h^ ≥3		0.85	0.56‐1.28	1.86	0.99‐3.50	1.18	0.80‐1.73	0.71	0.42‐1.19	1.28	0.87‐1.87
CHESS^i^ ≥3		0.71	0.32‐1.58	0.99	0.32‐3.09	2.14	1.09‐4.22^c^	0.28	0.06‐1.32	0.67	0.28‐1.59
Communication Scale ≥4		1.61	0.99‐2.62	0.99	0.42‐2.35	0.79	0.44‐1.43	1.07	0.56‐2.06	0.95	0.59‐1.53
RISE^j^ ≥3		1.22	0.72‐2.06	—	—	—	—	—	—	0.80	0.49‐1.30
Family support		0.74	0.41‐1.31	0.98	0.41‐2.35	1.28	0.70‐2.34	0.76	0.38‐1.52	0.59	0.35‐1.00^c^
Chewing problems		—	—	6.80	3.30‐14.03^k^	2.77	1.60‐4.78^k^	0.55	0.24‐1.31	1.20	0.66‐2.18
Discomfort or pain in the mouth		9.06	4.43‐18.54^k^	—	—	3.53	1.80‐6.90^k^	1.51	0.53‐4.25	3.18	1.37‐7.35^e^
Dry mouth		2.83	1.66‐4.83^k^	3.47	1.82‐6.65^k^	—	—	1.56	0.77‐3.16	1.21	0.68‐2.16
Gum problems		3.83	1.82‐8.06^k^	2.38	0.86‐6.58	0.71	0.30‐1.70	5.31	2.26‐12.44^k^	7.47	3.95‐14.14^k^
Tongue problems		0.83	0.33‐2.10	2.29	0.81‐6.51	2.56	1.19‐5.47^c^	6.06	2.64‐13.94^k^	3.24	1.29‐8.18^c^
Problems with palate and inner surface of cheeks and lips		1.07	0.41‐2.79	1.73	0.52‐5.79	2.51	1.01‐6.25^c^	0.75	0.21‐2.65	1.39	0.55‐3.54
Presence of teeth		0.65	0.43‐0.99^c^	0.66	0.35‐1.25	0.66	0.45‐0.98^c^	1.54	0.97‐2.45	—	—
Presence of dentures		0.67	0.43‐1.05	1.30	0.63‐2.69	0.70	0.45‐1.08	—	—	0.23	0.16‐0.33^k^
Modified mode of nutritional intake		8.77	5.60‐13.73^k^	0.38	0.15‐0.97^c^	0.78	0.43‐1.40	—	—	1.09	0.61‐1.96
Low weight (BMI 19)		2.02	1.12‐3.64^c^	—	—	1.80	1.02‐3.18^c^	—	—	1.48	0.84‐2.63
Polypharmacy (≥10 medications)		—	—	1.82	0.98‐3.36	1.47	1.00‐2.16^c^	—	—	—	—
Resistance to care		1.13	0.67‐1.92	—	—	0.49	0.26‐0.94^c^	1.38	0.73‐2.59	1.69	1.07‐2.66^c^
CAP^l^ behavior		—	—	1.39	0.69‐2.82	—	—	—	—	—	—
Reduced social interactions		—	—	1.24	0.63‐2.43	—	—	—	—	—	—
Severe vision impairment in adequate light		1.38	0.50‐3.79	—	—	—	—	1.30	0.30‐5.71	1.12	0.38‐3.29
Congestive heart failure		0.57	0.35‐0.93^c^	1.17	0.61‐2.25	1.33	0.89‐2.00	—	—	0.94	0.61‐1.45
Aspiration		2.38	1.35‐4.20^e^	—	—	2.28	1.21‐4.28^c^	—	—	1.69	0.85‐3.37
Pneumonia		2.07	0.52‐8.19	0.71	0.09‐5.28	1.63	0.51‐5.17	0.88	0.17‐4.45	1.41	0.41‐4.81
Diabetes		—	—	—	—	—	—	—	—	—	—
Reflux		—	—	—	—	2.56	1.20‐5.44^c^	—	—	—	—
COPD^m^		—	—	—	—	2.07	1.26‐3.41^e^	—	—	—	—
Parkinson disease		—	—	—	—	1.36	0.68‐2.71	—	—	—	—

a Data included in the model/total available oral health data.

b OR: odds ratio (significant OR >1: significant contributor; significant OR <1: significant protector).

c *P*<.05.

d ADLH: Activities of Daily Living Hierarchy.

e *P*<.01.

f Not included in the model.

g CPS: Cognitive Performance Scale.

h DRS: Depression Rating Scale.

i CHESS: Changes in Health, End-stage disease, Signs, and Symptoms.

j RISE: Revised Index of Social Engagement.

k *P*<.001.

l CAP: collaborative action point.

m COPD: chronic obstructive pulmonary disease.

**Table 5.** Factors associated with each oral health item (reference category: acceptable condition) in the adjusted logistic models.

		Teeth (895/1147)^a^		Gums (1657/2006)^a^		Tongue (1665/2057)^a^		Palate and inner surface of cheeks and lips (1605/1959)^a^		CAP^b^ oral hygiene (1629/2289)^a^		CAP referral to a dentist (2081/2221)^a^	
		OR^c^	95% CI	OR	95% CI	OR	95% CI	OR	95% CI	OR	95% CI	OR	95% CI
Age (years; 65-75: reference category)													
76‐85		1.72	1.10‐2.68^d^	1.06	0.61‐1.85	0.76	0.38‐1.50	0.68	0.33‐1.39	0.93	0.66‐1.32	0.91	0.70‐1.17
>85		1.51	0.87‐2.61	1.13	0.58‐2.19	1.11	0.52‐2.36	0.82	0.36‐1.85	0.83	0.55‐1.26	0.88	0.65‐1.20
Sex (male: reference category)													
Female		1.35	0.91‐1.98	1.17	0.72‐1.90	1.22	0.68‐2.19	1.25	0.66‐2.35	1.47	1.08‐1.99^d^	0.97	0.77‐1.22
Smoker		1.05	0.49‐2.25	0.92	0.36‐2.36	1.97	0.80‐4.88	1.59	0.61‐4.14	1.13	0.67‐1.91	1.03	0.69‐1.55
ADLH^e^ Scale ≥3		1.66	1.12‐2.47^d^	1.06	0.65‐1.73	1.55	0.84‐2.85	1.68	0.87‐3.25	—^f^	—	1.81	1.43‐2.30^g^
Extensive to total dependency for personal hygiene		—	—	—	—	—	—	—	—	1.63	1.20‐2.22^h^	—	—
CPS^i^ ≥3		0.82	0.54‐1.25	1.05	0.62‐1.77	1.12	0.59‐2.12	0.53	0.26‐1.11	1.07	0.77‐1.50	1.09	0.85‐1.41
DRS^j^ ≥3		1.14	0.76‐1.71	1.37	0.84‐2.24	1.14	0.65‐2.03	1.86	1.00‐3.47^d^	1.11	0.81‐1.53	1.27	1.02‐1.59^d^
CHESS^k^ ≥3		0.71	0.26‐1.90	0.41	0.12‐1.37	3.68	1.62‐8.37^h^	2.16	0.84‐5.54	0.77	0.37‐1.61	1.11	0.72‐1.72
Communication Scale ≥4		1.05	0.63‐1.76	0.99	0.53‐1.84	1.14	0.53‐2.43	1.54	0.67‐3.56	1.05	0.71‐1.57	1.35	1.03‐1.77^d^
RISE^l^ ≥3		1.04	0.63‐1.73	—	—	—	—	—	—	—	—	1.09	0.83‐1.43
Family support		0.52	0.30‐0.89^d^	0.59	0.32‐1.10	0.91	0.42‐2.00	0.56	0.26‐1.21	0.73	0.48‐1.12	0.68	0.50‐0.92^d^
Chewing problems		2.20	1.19‐4.07^d^	2.81	1.46‐5.39^h^	0.69	0.29‐1.67	1.62	0.69‐3.79	0.99	0.59‐1.66	—	—
Discomfort or pain in the mouth		2.02	0.78‐5.22	2.94	1.28‐6.72^d^	3.14	1.28‐7.70^d^	4.84	2.03‐11.54^g^	1.48	0.74‐2.95	—	—
Dry mouth		1.56	0.85‐2.86	1.16	0.58‐2.34	3.17	1.64‐6.14^g^	2.88	1.41‐5.86^h^	1.30	0.81‐2.08	—	—
Gum problems		7.23	3.92‐13.30^g^	—	—	5.76	2.73‐12.12^g^	—	—	8.08	4.66‐14.00^g^	—	—
Tongue problems		3.32	1.25‐8.80^d^	5.99	2.70‐13.29^g^	—	—	—	—	3.68	1.92‐7.06^g^	—	—
Problems with palate and inner surface of cheeks and lips		1.51	0.55‐4.12	—	—	—	—	—	—	1.00	0.45‐2.18	—	—
Presence of teeth		—	—	5.23	2.83‐9.66^g^	0.63	0.34‐1.16	1.81	0.92‐3.56	—	—	—	—
Presence of dentures		0.48	0.33‐0.70^g^	0.69	0.43‐1.11	0.63	0.33‐1.18	0.61	0.32‐1.17	—	—	—	—
Modified mode of nutritional intake		0.97	0.53‐1.75	1.12	0.56‐2.26	1.16	0.52‐2.58	0.77	0.31‐1.90	0.71	0.44‐1.14	3.47	2.64‐4.57^g^
Low weight (BMI ≤19)		—	—	—	—	—	—	1.22	0.50‐2.98	1.58	0.98‐2.55	1.35	0.95‐1.90
Polypharmacy (≥10 medications)		—	—	—	—	—	—	—	—	—	—	—	—
Resistance to care		1.83	1.13‐2.98^d^	1.45	0.82‐2.58	—	—	1.05	0.46‐2.36	1.47	0.99‐2.17	0.95	0.72‐1.25
CAP behavior		—	—	—	—	—	—	—	—	—	—	—	—
Reduced social interactions		—	—	1.01	0.60‐1.70	—	—	—	—	0.90	0.63‐1.27	—	—
Severe vision impairment in adequate light		0.67	0.19‐2.40	1.23	0.38‐4.01	3.66	1.25-10.73^d^	—	—	1.28	0.52‐3.13	1.29	0.75‐2.24
Congestive heart failure		—	—	1.24	0.74‐2.10	—	—	—	—	0.68	0.48‐0.99^d^	0.93	0.72‐1.20
Aspiration		—	—	1.06	0.44‐2.56	0.83	0.28‐2.48	—	—	—	—	1.50	1.04‐2.17^d^
Pneumonia		—	—	1.79	0.49‐6.56	1.56	0.29‐8.23	1.44	0.27‐7.67	1.30	0.45‐3.77	2.11	1.00‐4.44
Diabetes		—	—	0.79	0.44‐1.43	2.46	1.38‐4.36^h^	—	—	—	—	—	—
Reflux		—	—	—	—	—	—	—	—	—	—	—	—
COPD^m^		—	—	—	—	—	—	—	—	—	—	—	—
Parkinson disease		—	—	—	—	—	—	—	—	—	—	—	—

a Data included in the model/total available oral health data.

b CAP: collaborative action point.

c OR: odds ratio (significant OR >1: significant contributor; significant OR <1: significant protector).

d *P*<.05.

e ADLH: activities of daily living hierarchy.

f Not included in the model.

g *P*<.001.

h *P*<.01.

i CPS: Cognitive Performance Scale.

j DRS: Depression Rating Scale.

k CHESS: Changes in Health, End-stage disease, Signs, and Symptoms.

l RISE: Revised Index of Social Engagement.

m COPD: chronic obstructive pulmonary disease.

The following factors were significantly associated with *chewing problems*, after controlling for confounders: discomfort or pain in the mouth (OR 9.1, 95% CI 4.4‐18.5), modified mode of nutritional intake (OR 8.8, 95% CI 5.6‐13.7), gum problems (OR 3.8, 95% CI 1.8‐8.1), dry mouth (OR 2.8, 95% CI 1.7‐4.8), aspiration problems (OR 2.4, 95% CI 1.4‐4.2), ADLH problems (OR 2.2, 95% CI 1.3‐3.5), and low weight (OR 2.0, 95% CI 1.1‐3.6). This means that older adults with these conditions were more likely to have chewing difficulties than older adults with no such conditions. In addition, older age (more than 76 years) was also associated with more chewing problems (OR approximately 2.0, 95% CI 1.1‐3.4) than those aged 65‐75 years. The presence of teeth (OR 0.7, 95% CI 0.4‐1.0) contributed to a better chewing function. Having heart failure (OR 0.6, 95% CI 0.4‐0.9) was associated with fewer chewing problems.

In the adjusted logistic model for *discomfort or pain in the mouth*, chewing function (OR 6.8, 95% CI 3.3‐14.0) and dry mouth (OR 3.5, 95% CI 1.8‐6.7) had significant ORs, which means that residents with chewing difficulties and dry mouth had a higher chance of discomfort or pain in the mouth than those with no such conditions. A modified mode of nutritional intake (OR 0.4, 95% CI 0.2‐1.0) was associated with less discomfort or pain in the mouth.

Chewing difficulties (OR 2.8, 95% CI 1.6‐4.8) and discomfort or pain in the mouth (OR 3.5, 95% CI 1.8‐6.9) were also identified as associated factors for a *dry mouth*. In addition, reflux (OR 2.6, 95% CI 1.2‐5.4), poor conditions of the tongue (OR 2.6, 95% CI 1.2‐5.5) and palate and inner surface of cheeks and lips (OR 2.5, 95% CI 1.0‐6.3), aspiration problems (OR 2.3, 95% CI 1.2‐4.3), health instability derived from the CHESS Scale (OR 2.1, 95% CI 1.0‐4.2), COPD (OR 2.1, 95% CI 1.3‐3.4), and low weight (OR 1.8, 95% CI 1.0‐3.2) contributed to experiencing a dry mouth. Residents taking 10 or more medications (OR 1.5, 95% CI 1.0‐2.2) were also more likely to have a dry mouth. The presence of teeth (OR 0.7, 95% CI 0.5‐1.0), cognitive impairment (OR 0.5, 95% CI 0.4‐0.8), and resisting care (OR 0.5, 95% CI 0.3‐0.9) showed protective effects, reducing the experience of dry mouth.

Gum (OR 5.3, 95% CI 2.3‐12.4, and OR 7.5, 95% CI 4.0‐14.1, respectively) and tongue problems (OR 6.1, 95% CI 2.6‐13.9, and OR 3.2, 95% CI 1.3‐8.2, respectively) are both contributing factors to poor *denture hygiene* and poor *oral hygiene*. In the oral hygiene model, residents with discomfort or pain in the mouth (OR 3.2, 95% CI 1.4‐7.4), resistance to care (OR 1.7, 95% CI 1.1‐2.7), or extensive to total dependence on personal hygiene (OR 1.7, 95% CI 1.1‐2.4) were also more likely to have poor oral hygiene. It was found that males were more prone to poor oral hygiene than females (OR 1.6, 95% CI 1.1‐2.3). Family support (OR 0.6, 95% CI 0.4‐1.0) and the presence of dentures (OR 0.2, 95% CI 0.2‐0.3) contributed to acceptable oral hygiene. Despite its lack of significance in the bivariate analyses, pneumonia status was included in the adjusted logistic models for denture hygiene and oral hygiene based on evidence from previous research. However, the models yielded no significant contribution to poor denture or oral hygiene.

The highest ORs in the adjusted logistic model for *teeth* were found for the condition of the gums (OR 7.2, 95% CI 3.9‐13.3) and tongue (OR 3.3, 95% CI 1.3‐8.8). This means that older adults presenting gum or tongue problems were more likely to have dental problems than those with no such conditions. Chewing difficulties yielded an OR of 2.2 (95% CI 1.2‐4.1), indicating an association with poor condition of the teeth. When resistance to care (OR 1.8, 95% CI 1.1‐3.0) or extensive dependence on personal care (ADLH; OR 1.7, 95% CI 1.1‐2.5) was reported, the association with dental problems was significant. The same was true for adults aged 76‐85 years (OR 1.7, 95% CI 1.1‐2.7) compared with those aged 65‐75 years. On the contrary, family support (OR 0.5, 95% CI 0.3‐0.9) and the presence of dentures (OR 0.5, 95% CI 0.3‐0.7) showed protective effects.

*Gum* problems were more frequently reported when residents had problems with the tongue (OR 6.0, 95% CI 2.7‐13.3) or still had teeth (OR 5.2, 95% CI 2.8‐9.7). In addition to both being associated with dry mouth, discomfort or pain in the mouth (OR 2.9, 95% CI 1.3‐6.7) and chewing difficulties (OR 2.8, 95% CI 1.5‐5.4) were also associated with gum problems. Based on evidence from previous research, diabetes and heart failure were included as variables in the adjusted logistic model. However, these conditions were not significantly associated with gum problems.

Gum problems (OR 5.8, 95% CI 2.7‐12.1) also presented the highest OR in the adjusted logistic model for *tongue* problems, followed by health instability (OR 3.7, 95% CI 1.6‐8.4). Severe vision impairment in adequate light (OR 3.7, 95% CI 1.3‐10.7) and diabetes (OR 2.5, 95% CI 1.4‐4.4) were also identified as contributors to tongue problems. Dry mouth (OR 3.2, 95% CI 1.6‐6.1, and OR 2.9, 95% CI 1.4‐5.9, respectively) and discomfort or pain in the mouth (OR 3.1, 95% CI 1.3‐7.7, and OR 4.8, 95% CI 2.0‐11.5, respectively) were both associated with tongue problems and *palate and inner surface of cheeks and lips*. In addition, depressive symptoms increased the chance of problems with the palate and inner surface of cheeks and lips (OR 1.9, 95% CI 1.0‐3.5).

Gum problems (OR 8.1, 95% CI 4.7‐14.0) were a major contributor to the activation of the CAP *oral hygiene,* as well as tongue problems (OR 3.7, 95% CI 1.9‐7.1), highlighting the need for improved oral care. Extensive to total dependence on personal hygiene (OR 1.6, 95% CI 1.2‐2.2) and being male compared with being female (OR 1.5, 95% CI 1.1‐2.0) also increased the chance of activation of the CAP. The model showed that residents with heart failure (OR 0.7, 95% CI 0.5‐1.0) were less likely to need help with daily oral care.

The CAP *referral to a dentist* was more likely to be activated when residents had a modified mode of nutritional intake (OR 3.5, 95% CI 2.6‐4.6) or were dependent on personal care (ADLH; OR 1.8, 95% CI 1.4‐2.3). Aspiration (OR 1.5, 95% CI 1.0‐2.2), communication problems (OR 1.4, 95% CI 1.0‐1.8), and depressive symptoms (OR 1.3, 95% CI 1.0‐1.6) also increased the chance of referral to a dentist. Family support showed a protective effect in the activation of the CAP (OR 0.7, 95% CI 0.5‐0.9).

Smoking was included in each adjusted logistic model but did not significantly contribute to the presence of oral health problems. Similarly, pneumonia was included in all models except the one for teeth (not significant in bivariate analysis) but showed no association when adjusted for the other variables. Reflux, COPD, and Parkinson disease were conditions included only in the model for dry mouth, with reflux and COPD showing significant associations with dry mouth, while Parkinson disease did not.

## Discussion

### Principal Findings

This study examined the associations between oral health and general health outcomes based on interRAI assessments of nursing home residents in Flanders and the Netherlands, performed by their caregivers. This is the first study to explore these associations using the OHS, the optimized Oral Health Screener for use in the interRAI instruments.

Advancements in prevention, restorative techniques, and dental materials have enabled older adults to retain their natural dentition longer [29]. Despite changing patterns of tooth retention, tooth loss is still prevalent in the older population. Research indicates that most individuals aged 85 years and older in industrialized countries wear removable dentures, with the majority being completely edentulous [29]. In addition, nursing home residents tend to have fewer teeth than those living independently [29]. In this study, at least 42.0% (961/2301) of participating residents had lost all of their natural teeth and at least 65.0% (1499/2310) were wearing removable dentures. This finding aligns with the prevalence of edentulism (40.0%‐60.0%) and the use of removable dentures (40.0%‐85.0%) among nursing home residents in Europe, as reported in a systematic review by Janssens et al [30].

Natural teeth are much more complex and time-consuming to clean than an edentulous mouth with complete dentures [29]. Tooth loss is frequently the result of dental caries and gum inflammation, resulting from poor oral hygiene [31]. This study suggests that the combination of natural teeth and dentures is associated with better oral hygiene and condition of the teeth. However, the exact number of remaining teeth among the residents in this study was unknown. Poor brushing and inadequate cleaning of dentures allow the accumulation of plaque and food debris, increasing the risk of infections in the mouth [32]. The interrelated nature of the oral health problems identified in this study may be consistent with research showing that the microbial environment associated with poor hygiene affects not only teeth and gums but also the entire oral cavity including the tongue, palate, and inner surface of cheeks and lips [33,34]. These areas may be affected by conditions such as ulcers, fungal infections such as candidiasis, or mucosal irritation [32,35]. Wong et al [36] confirmed that poor oral hygiene among residents was probably due to inadequate brushing or cleaning of teeth and dentures, which increases the prevalence of dental problems, periodontal diseases, and other severe complications.

Age-related conditions, such as functional impairment, frailty, and chronic illnesses, reduce residents’ ability to self-care and increase their reliance on caregivers for their daily care [3,5,37]. Such dependence on others for personal care was associated with a higher risk of chewing difficulties, poor condition of the teeth, and a higher need for dental referral. The increased retention of natural teeth, along with the growing need for care and support in old age, may explain why significantly more dental problems were observed in adults aged 76‐85 years than in those a decade younger [29,38]. In addition, extensive to total dependence on personal hygiene was associated with poor oral hygiene and activation of the CAP oral hygiene, consistent with previous research on oral health among older institutionalized residents, identifying high care dependency as a predisposing factor for poor oral hygiene [36]. The association was particularly true for men compared with women and aligns with broader research indicating that women tend to have better oral health behaviors [39]. However, oral care is often neglected when assistance is needed for activities of daily living [29]. Research has shown that caregivers often lack knowledge of oral health, give lower priority to oral care over other care tasks, and consider the mouth as an intimate area of the body [15]. In addition, residents’ resistance to care was often associated with oral health problems, as confirmed by this study [15]. Residents may also lack awareness of oral health problems due to health conditions such as cognitive impairment, sensory losses (eg, impaired vision or reduced sense of taste and smell), or the misconception that poor oral health is part of aging [3,37,40,41]. This may explain the association between severe vision impairment in adequate light and more tongue problems.

A higher need for dental referral was found when residents struggled to express themselves or understand others, which is consistent with previous research that suggests that nonverbal individuals may be more vulnerable to oral health problems [42]. Research suggests that older adults often visit the dentist only in the event of certain symptoms, such as pain, rather than regularly for preventive care [36]. As they are unable to communicate discomfort or pain, it can be challenging for caregivers to address these problems on time [42]. This highlights the need for targeted training and support for caregivers to address these challenges and ensure better oral health outcomes for residents [43]. Family support was identified as beneficial for oral health outcomes. Family members can assist with daily oral hygiene, or can ensure or demand consistent care, as well as bridge gaps in communication or resistance [44].

This study showed that the presence of teeth plays an important role in residents’ ability to chew all types of food. The scoping review by Lahoud et al [8] demonstrated that tooth loss and musculoskeletal conditions, including frailty and sarcopenia—age-related loss of skeletal muscle mass and function, are the most common factors that negatively affect chewing function. Chewing difficulties are associated with discomfort or pain in the mouth and can negatively affect food selection and nutritional intake, increasing the risk of malnutrition [8]. This study confirmed that the type of food consumed may also contribute to chewing difficulties. Diets that primarily consist of softer foods can lead to a decline in chewing function due to reduced use of the masticatory muscles and teeth. Furthermore, insufficient intake of essential nutrients, such as folic acid and vitamin A, may exacerbate oral health problems, including chewing difficulties [8,45]. Consistent with the systematic review by Fan et al [46], this study identified discomfort or pain in the mouth, dry mouth, and periodontal diseases as contributing to chewing problems, underscoring the importance of maintaining good oral health to support proper chewing. Interestingly, individuals with heart failure experienced fewer chewing difficulties, which could result from lifestyle changes, including dietary adjustments or more frequent medical consultations. However, further research is needed to clarify these associations and explore underlying mechanisms.

Significant associations were found between chewing function, discomfort or pain in the mouth, and dry mouth. Chewing stimulates the salivary glands, enhancing the production of saliva, which facilitates the swallowing of food [47,48]. Lack of saliva can lead to dry mouth, which can cause discomfort or pain in the mouth and make it difficult to chew food, often necessitating dietary adjustments [47]. The association between the presence of teeth and residents experiencing less dry mouth is consistent with research showing that individuals with natural teeth generally retain a more effective chewing function than those with complete dentures, as dentures alone do not provide the same mechanical stimulation to the salivary glands [49]. Saliva also contributes to the protection of oral mucosa from pathogens [48]. The latter could explain the association between dry mouth and poor condition of the tongue and palate and the inner surface of cheeks and lips. In addition, it is also important to note that in the earlier version of the OHS, the ohr-interRAI, the item Tongue received a negative (poor) score when a dry tongue was observed [17]. Since the adjustment of the item was made in 2023 and the assessments were collected between 2020 and 2024, it is possible that caregivers took this into account when assessing the mouth [18].

Dry mouth is a common side effect of medication, particularly among older adults, who often take multiple medications due to a range of chronic systemic diseases [50]. This may explain the association between health instability and dry mouth, in addition to dehydration—one of the items in the CHESS Scale indicating health instability and a known cause of dry mouth [51]. Reflux and COPD were also associated with dry mouth. Reflux may lead to dry mouth due to acid exposure, which irritates salivary glands, reducing their function [52]. Residents with COPD may experience dry mouth as a side effect of inhaled medications or due to chronic mouth breathing, which exacerbates dryness [53]. However, further research is necessary to clarify the underlying mechanisms. Interestingly, this study suggested that dry mouth is linked to better cognitive function, which may be explained by medications commonly prescribed for dementia that improve cognition while also causing dry mouth as a side effect.

This study yielded significant associations between depressive symptoms and poor conditions of the palate and inner surface of cheeks and lips, as well as an increased need for dental referrals. Antidepressants, commonly prescribed for the management of depression and other mental health problems, are known to increase the likelihood of dry mouth, increasing the prevalence of oral health problems, including mouth sores and cracked lips [50,54]. In addition, the associations may be explained by research indicating that individuals with depressive symptoms often experience a lack of motivation and energy, which may lead to neglect of oral hygiene practices, contributing to poor oral health outcomes [55,56]. Several studies confirm the link between depressive symptoms and poor oral health outcomes among older adults [57-59]. Although depression is not a normal part of aging, older adults—particularly nursing home residents compared with those living independently—are more likely to experience loneliness and social isolation, poor physical health, and restrictions in daily activities, which can increase the risk of depression [60]. Given the high prevalence of depressive symptoms and oral health problems in nursing home residents, timely identification and management of both conditions is important due to their bidirectional relationship [1,60,61].

Despite research showing evidence on the relationship between periodontitis and systemic diseases such as cardiovascular disease and diabetes, these diseases were not significantly linked to gum problems in this study [3]. This discrepancy may be due to methodological factors, such as the sample size, the participants included, or the assessments by the caregivers. Diabetes was, however, associated with tongue problems. Diabetes is common in nursing home residents, with reported prevalence rates ranging from 20.0% to 34.0%, aligning with the prevalence of 21.6% (506/2344) in this study [62]. This condition is well known for its association with oral health problems, including an increased risk of developing oral infections and alterations to the oral mucosa, such as a coated and fissured tongue or aphthous stomatitis [63].

To better understand the results of this study, some key considerations and limitations should be acknowledged. The generalizability of the results to the wider population is limited. All participants resided in nursing homes where a specific software system was used, integrating the OHS into the interRAI instrument. While using the interRAI instrument became mandatory in Flemish nursing homes since June 2023, this was not the case in the Netherlands. The Dutch participating nursing homes, all part of the Omring group, specifically requested to use the interRAI LTCF, including the OHS [64]. It is therefore possible that these homes were particularly motivated to work on improving residents’ oral care and oral health, which may have impacted the results, in addition to possible differences in context and health practices between Flanders and the Netherlands. Schoebrechts et al [19] compared the oral health status of older adults living in Flemish and Dutch nursing homes assessed by nondental caregivers using the OHS and found more oral health problems among Flemish than Dutch residents. In addition, they reported some differences in general health status, including a higher level of dependence on others for activities of daily living and personal hygiene, as well as fewer depressive symptoms among Flemish residents compared with their Dutch counterparts [19]. Comparing associations between oral health and general health outcomes among nursing home residents in Flanders and the Netherlands was beyond the scope of this study but could be a subject for future research.

The quality and accuracy of the interRAI assessments also depend on the knowledge and qualifications of the caregivers completing them. Previous research has shown that nondental caregivers often underestimate oral health problems in nursing home residents compared with dental professionals [17]. This underestimation may be due to lack of knowledge, inadequate training, limited experience, or discomfort in addressing oral health [15,17]. In addition, underestimation of oral health problems may be attributed to some assessment criteria. For instance, oral and denture hygiene are assessed based on the extent of plaque accumulation, with the presence of plaque covering more than one-third of a tooth or denture surface. It should be noted that this may be a crude measure for the assessors. Another reason may be due to limitations of the observational assessment, particularly when residents have communication difficulties, cognitive impairment, or resist care.

In this study, the oral health assessments using the OHS were performed for the first time by various caregivers whose training or education was unknown. Therefore, it can be assumed that oral health problems identified through the assessment may have been severe. This highlights the importance of regular oral health assessments by caregivers to identify oral health problems that may otherwise go unnoticed, as well as the need for adequate training and practice.

Some of the associations that are observed in this study can be deduced from logical connections or previous research. Other associations remain less well explained, as they appear to be nonlogical at first glance or contradict existing studies. Therefore, caution is warranted when interpreting the results. Further research may explore these associations more thoroughly, contributing to a better understanding of the relationship between oral and general health in nursing home populations. Given the mandatory use of the interRAI instrument in nursing homes in Flanders, analyses of larger datasets can also facilitate the determination of the validity of the results and how they sustain over time. However, it should be noted that a large sample size can lead to statistically significant findings that do not necessarily have clinical significance [65]. In addition, it is important to note that the interRAI instrument primarily records the occurrence of conditions. Further research is needed to identify causal factors and inform targeted interventions.

### Strengths

Collecting data with the interRAI instrument can be considered as a strength of this study as it is a validated, comprehensive, holistic, and widely used tool for assessing residents’ health and well-being. In addition, the integration of the OHS showed significant improvements over its current version, allowing for more accurate identification of oral health problems [66].

### Conclusions

This study provided valuable insights into the associations between oral health and general health outcomes, underscoring the importance of a holistic approach that considers oral health as an integral part of general health and well-being. Recognizing these associations can enhance care planning and improve quality of care.

In practical terms, these findings support measures such as integrating regular oral health assessments into care protocols, ensuring timely access to oral health professionals, and providing caregivers with targeted training on oral care and identifying poor oral health. In addition, the results showed the importance of interdisciplinary collaboration to comprehensively address oral health. An integrated approach has the potential to improve both oral and general health outcomes, help reduce the risk of related complications, and ultimately enhance residents’ quality of life.

## Supplementary material

10.2196/72308 Multimedia Appendix 1 Factors associated with each oral health item (reference category: acceptable condition) in the bivariate analyses.

## Abbreviations

ADLH: Activities of Daily Living Hierarchy
CAP: collaborative action point
CHESS: Changes in Health, End-Stage Disease, Signs, and Symptoms
COPD: chronic obstructive pulmonary disease
CPS: Cognitive Performance Scale
DRS: Depression Rating Scale
LTCF: long-term care facility
OR: odds ratio
