# Supplementary material for: Insights Into Associations Between Oral and General Health Outcomes of Nursing Home Residents Based on InterRAI Data: Cross-Sectional Study
Source: JMIR Aging. 2025 Oct 17;8:e72308. doi: 10.2196/72308 (PMC12533934; doi:10.2196/72308)
Supplement: Multimedia Appendix 1 [file aging-v8-e72308-s001.docx]

## Multimedia Appendix 1

**Table S1.** Factors associated with each oral health item (ref. category: acceptable condition) in the bivariate analyses.^a,b^

|  | **Chewing function** | | **Discomfort or pain in the mouth** | | **Dry mouth** | | **Denture hygiene** | | **Oral hygiene** | | **Teeth** | | **Gums** | | **Tongue** | | **Palate and inner surface of cheeks and lips** | | **CAP oral hygiene** | | **CAP referral to a dentist** | |
| --- | --- | --- | --- | --- | --- | --- | --- | --- | --- | --- | --- | --- | --- | --- | --- | --- | --- | --- | --- | --- | --- | --- |
|  | Corr. Value | *P*-value | Corr. Value | *P*-value | Corr. Value | *P*-value | Corr. Value | *P*-value | Corr. Value | *P*-value | Corr. Value | *P*-value | Corr. Value | *P*-value | Corr. Value | *P*-value | Corr. Value | *P*-value | Corr. Value | *P*-value | Corr. Value | *P*-value |
| Age | 0.053 | .01 | 0.006 | .76 | 0.022 | .30 | -0.038 | .15 | -0.075 | .004 | -0.026 | .39 | -0.039 | .08 | 0.002 | .94 | -0.028 | .22 | -0.061 | .003 | -0.017 | .43 |
| Gender | *-0.054* | .01 | *-0.015* | .48 | *-0.037* | .08 | *0.047* | .07 | *0.058* | .02 | *0.052* | .08 | *0.027* | .23 | *-0.002* | .94 | *0.015* | .50 | *0.051* | .02 | *-0.012* | .58 |
| Smoker | *-0.026* | .22 | *-0.008* | .70 | *0.012* | .57 | *0.013* | .63 | *0.013* | .61 | *0.029* | .32 | *-0.022* | .32 | *0.010* | .65 | *0.007* | .74 | *-0.001* | .96 | *-0.018* | .39 |
| ADLH scale ≥ 3 | *0.215* | <.001 | *0.022* | .30 | *0.052* | .01 | *0.019* | .47 | *0.155* | <.001 | *0.141* | <.001 | *0.073* | .001 | *0.078* | <.001 | *0.074* | .001 | *0.098* | <.001 | *0.222* | <.001 |
| CPS ≥ 3 | *0.160* | <.001 | *0.022* | .29 | *-0.085* | <.001 | *0.020* | .44 | *0.112* | <.001 | *0.093* | .002 | *0.063* | .005 | *0.022* | .33 | *0.014* | .53 | *0.061* | .004 | *0.122* | <.001 |
| DRS ≥ 3 | *0.056* | .008 | *0.106* | <.001 | *0.032* | .14 | *0.009* | .74 | *0.105* | <.001 | *0.080* | .007 | *0.071* | .002 | *0.044* | .04 | *0.068* | .003 | *0.063* | .003 | *0.077* | <.001 |
| CHESS ≥ 3 | *0.097* | <.001 | *0.079* | <.001 | *0.109* | <.001 | *-0.043* | .10 | *0.030* | .25 | *0.048* | .11 | *0.056* | .01 | *0.131* | <.001 | *0.096* | <.001 | *0.011* | .61 | *0.088* | <.001 |
| Communication scale ≥ 4 | *0.222* | <.001 | *0.028* | .18 | *-0.032* | .14 | *0.020* | .45 | *0.111* | <.001 | *0.107* | <.001 | *0.106* | <.001 | *0.059* | .007 | *0.071* | .002 | *0.062* | .003 | *0.180* | <.001 |
| RISE ≥ 3 | *-0.064* | .003 | *-0.028* | .19 | *0.007* | .76 | *-0.016* | .53 | *-0.056* | .03 | *-0.072* | .02 | *-0.042* | .06 | *-0.017* | .45 | *-0.027* | .24 | *-0.026* | .22 | *-0.057* | .007 |
|  |  |  |  |  |  |  |  |  |  |  |  |  |  |  |  |  |  |  |  |  |  |  |
| Chewing problems |  |  | *0.202* | <.001 | *0.147* | <.001 | *0.021* | .43 | *0.170* | <.001 | *0.213* | <.001 | *0.150* | <.001 | *0.075* | <.001 | *0.109* | <.001 | *0.085* | <.001 | *0.624* | <.001 |
| Discomfort or pain in the mouth | *0.202* | <.001 |  |  | *0.189* | <.001 | *0.056* | .03 | *0.138* | <.001 | *0.172* | <.001 | *0.157* | <.001 | *0.120* | <.001 | *0.142* | <.001 | *0.087* | <.001 | *0.344* | <.001 |
| Dry mouth | *0.147* | <.001 | *0.189* | <.001 |  |  | *0.039* | .14 | *0.083* | .001 | *0.134* | <.001 | *0.087* | <.001 | *0.135* | <.001 | *0.126* | <.001 | *0.058* | .008 | *0.210* | <.001 |
| Poor denture hygiene | *0.021* | .43 | *0.056* | .03 | *0.039* | .14 |  |  | *0.478* | <.001 | *0.336* | <.001 | *0.235* | <.001 | *0.189* | <.001 | *0.111* | <.001 | *0.785* | <.001 | *0.241* | <.001 |
| Poor oral hygiene | *0.170* | <.001 | *0.138* | <.001 | *0.083* | .001 | *0.478* | <.001 |  |  | *0.551* | <.001 | *0.430* | <.001 | *0.194* | <.001 | *0.238* | <.001 | *0.927* | <.001 | *0.502* | <.001 |
| Tooth problems | *0.213* | <.001 | *0.172* | <.001 | *0.134* | <.001 | *0.336* | <.001 | *0.551* | <.001 |  |  | *0.423* | <.001 | *0.172* | <.001 | *0.269* | <.001 | *0.490* | <.001 | *0.770* | <.001 |
| Gum problems | *0.150* | <.001 | *0.157* | <.001 | *0.087* | <.001 | *0.235* | <.001 | *0.430* | <.001 | *0.423* | <.001 |  |  | *0.217* | <.001 | *0.453* | <.001 | *0.382* | <.001 | *0.444* | <.001 |
| Tongue problems | *0.075* | <.001 | *0.120* | <.001 | *0.135* | <.001 | *0.189* | <.001 | *0.194* | <.001 | *0.172* | <.001 | *0.217* | <.001 |  |  | *0.374* | <.001 | *0.170* | <.001 | *0.326* | <.001 |
| Problems with alate and inner surface of cheeks and lips | *0.109* | <.001 | *0.142* | <.001 | *0.126* | <.001 | *0.111* | <.001 | *0.238* | <.001 | *0.269* | <.001 | *0.453* | <.001 | *0.374* | <.001 |  |  | *0.190* | <.001 | *0.316* | <.001 |
| CAP Oral hygiene | *0.085* | <.001 | *0.087* | <.001 | *0.058* | .008 | *0.785* | <.001 | *0.927* | <.001 | *0.490* | <.001 | *0.382* | <.001 | *0.170* | <.001 | *0.316* | <.001 |  |  | *0.390* | <.001 |
| CAP Referral to a dentist | *0.624* | <.001 | *0.344* | <.001 | *0.210* | <.001 | *0.241* | <.001 | *0.502* | <.001 | *0.770* | <.001 | *0.444* | <.001 | *0.326* | <.001 | *0.190* | <.001 | *0.390* | <.001 |  |  |
| Presence of teeth | *-0.055* | .01 | *-0.001* | .96 | *-0.025* | .26 | *0.054* | .04 |  |  |  |  | *-0.159* | <.001 | *-0.014* | .52 | *0.058* | .01 |  |  |  |  |
| Presence of dentures | *-0.101* | <.001 | *-0.005* | .83 | *-0.015* | .50 |  |  | *-0.304* | <.001 | *-0.210* | <.001 | *0.185* | <.001 | *-0.072* | .001 | *-0.101* | <.001 |  |  |  |  |
|  |  |  |  |  |  |  |  |  |  |  |  |  |  |  |  |  |  |  |  |  |  |  |
| Anxiety | *0.102* | <.001 | *0.107* | <.001 | *0.051* | .02 | *0.033* | .20 | *0.071* | .006 | *0.048* | .10 | *0.039* | .08 | *0.003* | .90 | *0.058* | .01 | *0.038* | .07 | *0.097* | <.001 |
| Schizophrenia | *0.056* | .008 | *0.071* | <.001 | *-0.005* | .83 | *-0.031* | .24 | *0.023* | .36 | *0.039* | .19 | *0.022* | .33 | *0.003* | .89 | *0.027* | .23 | *-0.006* | .79 | *0.052* | .02 |
| Bipolar | *0.064* | .002 | *-0.004* | .86 | *0.027* | .20 | *-0.018* | .49 | *-0.005* | .85 | *0.016* | .59 | *-0.001* | .97 | *-0.004* | .85 | *-0.003* | .91 | *-0.015* | .48 | *0.033* | .12 |
| Pneumonia | *0.053* | .01 | *0.042* | .05 | *0.058* | .007 | *0.041* | .12 | *0.046* | .08 | *0.024* | .41 | *0.075* | <.001 | *0.065* | .003 | *0.091* | <.001 | *0.039* | .07 | *0.043* | .04 |
| Aspiration | *0.237* | <.001 | *0.023* | .27 | *0.092* | <.001 | *0.028* | .28 | *0.098* | <.001 | *0.028* | .34 | *0.093* | <.001 | *0.074* | <.001 | *0.041* | .07 | *0.057* | .006 | *0.172* | <.001 |
| Reflux | *0.000* | .98 | *0.029* | .17 | *0.075* | <.001 | *-0.013* | .61 | *0.024* | .35 | *-0.020* | .51 | *0.032* | .15 | *0.004* | .84 | *-0.006* | .78 | *0.004* | .83 | *0.010* | .65 |
| Dehydration | *0.068* | .001 | *0.026* | .22 | *0.081* | <.001 | *-0.030* | .24 | *0.036* | .16 | *-0.006* | .85 | *0.017* | .45 | *0.107* | <.001 | *0.073* | .001 | *0.006* | .79 | *0.070* | <.001 |
| Low weight (BMI ≤ 19) | *0.114* | <.001 | *0.008* | .72 | *0.044* | .04 | -0.014 | .61 | *0.090* | <.001 | 0.009 | .76 | *0.007* | .78 | *0.028* | .22 | *0.061* | .008 | *0.065* | .002 | *0.091* | <.001 |
| Dementia | *0.063* | .003 | *0.026* | .22 | *-0.039* | .07 | *0.021* | .43 | *0.005* | .85 | *0.033* | .26 | *0.033* | .14 | *0.024* | .28 | *0.051* | .02 | *-0.007* | .73 | *0.053* | .01 |
| Diabetes mellitus | *-0.037* | .08 | *0.006* | .79 | *0.030* | .16 | *0.012* | .63 | *-0.005* | .86 | *0.018* | .55 | *-0.031* | .17 | *0.045* | .04 | *-0.040* | .08 | *-0.013* | .54 | *-0.005* | .80 |
| Parkinson | *0.040* | .06 | *0.002* | .91 | *0.061* | .004 | *-0.022* | .40 | *-0.015* | .57 | *-0.029* | .33 | *-0.005* | .81 | *0.012* | .60 | *-0.001* | .97 | *-0.015* | .48 | *0.033* | .12 |
| COPD | *-0.033* | .12 | *0.038* | .07 | *0.095* | <.001 | *-0.033* | .21 | *-0.020* | .44 | *-0.013* | .66 | *0.013* | .56 | *0.026* | .23 | *-0.008* | .73 | *-0.029* | .16 | *-0.018* | .40 |
| Congestive heart failure | *-0.049* | .02 | *0.061* | .004 | *0.063* | .003 | *-0.019* | .47 | *-0.060* | .02 | *-0.018* | .54 | *-0.020* | .36 | *0.023* | .30 | *-0.017* | .46 | *-0.057* | .007 | *-0.043* | .04 |
| Cancer | *0.009* | .67 | *0.041* | .06 | *0.003* | .88 | *-0.006* | .82 | *-0.023* | .37 | *0.001* | .97 | *-0.026* | .24 | *0.017* | .46 | *-0.009* | .70 | *-0.036* | .09 | *0.000* | .99 |
|  |  |  |  |  |  |  |  |  |  |  |  |  |  |  |  |  |  |  |  |  |  |  |
| Polypharmacy (≥ 10 medications) | *-0.004* | .83 | *0.074* | <.001 | *0.103* | <.001 | *0.015* | .57 | *-0.024* | .35 | *0.038* | .20 | *0.040* | .07 | *0.032* | .15 | *0.027* | .23 | *0.003* | .88 | *0.033* | .12 |
| Modified mode of nutritional intake | *0.481* | <.001 | *0.060* | .005 | *0.076* | <.001 | *-0.002* | .93 | *0.154* | <.001 | *0.132* | <.001 | *0.092* | <.001 | *0.073* | <.001 | *0.055* | .02 | *0.060* | .004 | *0.327* | <.001 |
| Behavior problems | *0.107* | <.001 | *0.073* | <.001 | *-0.024* | .27 | *0.062* | .02 | *0.138* | <.001 | *0.167* | <.001 | *0.084* | <.001 | *0.042* | .06 | *0.076* | <.001 | *0.087* | <.001 | *0.116* | <.001 |
| Resistance to care | *0.092* | <.001 | *0.022* | .30 | *-0.077* | <.001 | *0.064* | .01 | *0.127* | <.001 | *0.128* | <.001 | *0.071* | .002 | *-0.007* | .77 | *0.064* | .005 | *0.077* | <.001 | *0.068* | .001 |
| Extensive to total dependency for personal hygiene | 0.235 | <.001 | 0.031 | .15 | 0.038 | .08 | *0.029* | .27 | *0.159* | <.001 | 0.150 | <.001 | 0.090 | <.001 | 0.083 | <.001 | 0.068 | .003 | *0.102* | <.001 | *0.239* | <.001 |
| Severe vision impairment in adequate light | *0.058* | .006 | *0.018* | .40 | *0.012* | .57 | *0.054* | .04 | *0.074* | .004 | *0.066* | .03 | *0.056* | .01 | *0.049* | .03 | *0.016* | .49 | *0.053* | .01 | *0.058* | .007 |
| Reduced social interactions | *0.060* | .004 | *0.055* | .01 | *-0.007* | .75 | *-0.015* | .57 | *0.090* | <.001 | *0.114* | <.001 | *0.057* | .01 | *0.005* | .83 | *0.006* | .78 | *0.043* | .04 | *0.097* | <.001 |
| Strong and supportive relationship with family | *-0.070* | <.001 | *-0.021* | .32 | *-0.019* | .37 | *-0.056* | .03 | *-0.127* | <.001 | *-0.157* | <.001 | *-0.095* | <.001 | *-0.057* | .01 | *-0.084* | <.001 | *-0.077* | <.001 | *-0.097* | <.001 |
|  |  |  |  |  |  |  |  |  |  |  |  |  |  |  |  |  |  |  |  |  |  |  |
| Physician visit in the last 14 days | -0.021 | .33 | 0.013 | .56 | 0.038 | .08 | -0.012 | .66 | 0.016 | .55 | 0.007 | .82 | 0.034 | .13 | 0.018 | .43 | 0.000 | .99 | 0.003 | .87 | 0.030 | .16 |
| Dental check-up in last year | *-0.012* | .56 | *0.036* | .09 | *-0.037* | .09 | *-0.034* | .19 | *-0.021* | .42 | *-0.029* | .32 | *0.044* | .05 | *-0.042* | .06 | *-0.002* | .92 | *-0.008* | .71 | *-0.025* | 0.236 |

^a^Green: significant contributor

^b^Blue: significant protector
